# Comparison of Recurrent and Naïve Keratitis in a Cohort of 1303 Patients

**DOI:** 10.3390/jcm14113760

**Published:** 2025-05-27

**Authors:** Maciej Kwiatkowski, Emilia Babula, Aleksandra Sikora, Justyna Izdebska, Janusz Skrzypecki, Jacek P. Szaflik, Joanna Przybek-Skrzypecka

**Affiliations:** 1 Department of Ophthalmology, Medical University of Warsaw, Sierakowskiego 13, 01-756 Warsaw, Poland; maciejkwiatkowski43@gmail.com (M.K.); ebabula99@gmail.com (E.B.); sikora.alk@gmail.com (A.S.); justyna.izdebska@wum.edu.pl (J.I.); jacek.szaflik@wum.edu.pl (J.P.S.); 2 Samodzielny Publiczny Kliniczny Szpital Okulistyczny, ul. Sierakowskiego 13, 03-709 Warsaw, Poland; 3 Department of Experimental Physiology and Pathophysiology, Medical University of Warsaw, 02-091 Warsaw, Poland; janusz.skrzypecki@wum.edu.pl

**Keywords:** keratitis, recurrent keratitis, naïve keratitis, legal blindness, therapeutic corneal transplant

## Abstract

**Objectives**: Microbial keratitis is a precursor to corneal scarring, the fifth-leading cause of blindness and visual impairment worldwide. Despite its significance, there is a paucity of data quantifying the recurrence rates of keratitis and the subsequent corneal damage. This study aims to address this gap by providing a comprehensive analysis of the frequency and origin of keratitis recurrences and its impact on visual deterioration. **Methods**: We retrospectively analyzed a cohort of 1303 patients diagnosed with microbial keratitis at the Independent Public University Eye Hospital in Warsaw, Poland, between January 2018 and December 2023. All adult patients with at least one documented episode of infectious keratitis were included in the study. Patients were divided into two cohorts: Group 1: patients with recurrent keratitis (n = 233) and Group 2: patients with the first episode of keratitis (n = 1070). **Results**: The recurrence rate of keratitis was 17.88% (233 of 1303 patients), regardless of etiology. Visual acuity on admission did not differ significantly between the recurrent and naïve groups. However, among patients with at least three episodes, visual acuity was worse (median logMAR for 1 vs. 2 vs. 3 recurrences were the following: 0.30 (0.08–0.67) vs. 0.60 (0.12–1.30) vs. 0.44 (0.20–0.92), accordingly, *p* = 0.049). Univariate logistic regression identified contact lens usage as a significant risk factor for recurrence (OR 2.37, 95% CI: 1.84–3.08, *p* < 0.001), also including its inappropriate use (OR 2.25, 95% CI: 1.42–3.66, *p* = 0.001). In terms of etiology, bacteria were the most common identified cause of keratitis in both the recurrent and naïve groups, accounting for 38.36% (90 cases) in Group 1 and 47% (503 cases) in Group 2. Viruses were the second most prevalent etiology, representing 31.33% (73 cases) in Group 1 and 19.91% (213 cases) in Group 2. Furthermore, a multivariate logistic regression model indicated that advanced age, delayed treatment, fungal etiology, and post-corneal transplant status were strongly associated with worse visual outcomes. **Conclusions**: Although each recurrence contributes to cumulative vision loss, the majority of patients with recurrent keratitis present with a useful visual acuity (0.3 to 0.60 logMAR). Our study identified older age, fungal etiology, delayed treatment, and post-keratoplasty keratitis as the most significant risk factors for visual deterioration. These findings underscore the need for targeted interventions in populations at higher risk of adverse visual outcomes.

## 1. Introduction

Microbial keratitis (MK) is a major cause of ocular morbidity, frequently resulting in significant discomfort and impairment of daily functioning, especially in cases of recurrence. Recurrent episodes often necessitate more intensive and prolonged treatment regimens, which may include surgical intervention [1]. The need for chronic preventative care further impacts an individual’s overall health and quality of life (QOL) [2]. Noteworthy, individuals who experienced keratitis with complete recovery of visual acuity scored lower on QOL assessments compared to unaffected individuals [3]. Similarly, research on viral keratitis revealed that even the latent phase of the disease could diminish QOL, comparable to other sight-threatening conditions [4]. Currently, the only approved preventative therapy targets viral herpetic keratitis, but it does not ensure complete therapeutic success [4]. Therefore, a deeper understanding of the mechanisms behind recurrent flare-ups is critical to improve both prevention and management strategies. The growing prevalence of multidrug-resistant microorganisms further complicates treatment, often resulting in more severe scarring and poorer outcomes. While several alternative strategies have proven their efficacy preclinically in multidrug-resistant keratitis (including formaldehyde-releasing agents or super-cationic carbon quantum dots synthesized from spermidine or cross-linking), they still require validation through human clinical trials [5,6,7].

Annual incidence rates of keratitis range from 27.6 to 40.3 cases per 100,000 people, varying between the world’s regions with distinct healthcare access and hygiene standards [2,8]. High-income countries such as the United States report incidence rates of 2.5–27.6 per 100,000 population–year [9,10], England—2.6–40.3 per 100,000 population–year [11,12,13], and Australia—6.6 per 100,000 population–year [14]. Notably, emerging economies with limited healthcare infrastructure report significantly higher incidence rates. For example, in the Madurai district of India, incidence of microbial keratitis is estimated at 113 per 100,000 population–year [15] or in Nepal at 799 per 100,000 population–year [16]. While the proportion of keratitis relapse in the general population remains unclear, viral keratitis studies estimate that 40% of patients experience 2–5 relapses, and 11% endure 6–15 episodes over their lifetime [17]. Microbial keratitis begins with the disruption of the corneal epithelial barrier, allowing pathogens to adhere to and invade the underlying tissue [18]. Once inside, microbes release enzymes and toxins that damage corneal cells and trigger a host immune response, leading to inflammation, tissue destruction, and potential scarring [19,20]. Nominally, each recurrence contributes to progressive corneal opacity and scarring, which causes 1.9 million cases of blindness worldwide annually, often necessitating corneal grafts [21,22]. Even though keratoplasty is considered a last-resort treatment, it is far from ideal. Failure rates in transplanted corneas range from 2 to 3% to as high as 41%, thereby perpetuating a vicious cycle of recurrence and intervention [23].

Although a substantial body of evidence exists on infectious keratitis, there is a notable gap in large-scale cohort studies specifically focused on recurrent keratitis, particularly within European populations. There is a pressing need for further investigation into the clinical course of keratitis, its potential for recurrence, and the relationship between its underlying etiology and patient outcomes. A deeper understanding of these factors would not only support a more personalized approach to patient care—enabling more effective treatments and faster recovery—but also help identify key risk factors that contribute to the recurrence rate. To address this issue, our study investigates the recurrence rate of microbial keratitis, as well as the demographic and clinical characteristics of patients with recurrent keratitis. We also examine whether visual outcomes differ significantly between patients with recurrent and naïve keratitis. Our findings aim to enhance patient counseling, improve clinical prognostication, and inform future research directions.

## 2. Methods

### 2.1. Study Participants

This retrospective, observational study included 1303 patients diagnosed with keratitis who were treated at the Emergency Eye Department of the Department of Ophthalmology, Medical University of Warsaw, between January 2018 and December 2023. Of these, 1106 patients were diagnosed and treated solely at the Emergency Department, while 197 patients were admitted to the hospital.

### 2.2. Data Collection

Data were extracted from the institutional database of the SPKSO Ophthalmic University Hospital in Warsaw, Poland, covering the period from January 2018 to December 2023. Patient selection was based on the International Classification of Diseases, 10th Revision (ICD-10), using diagnostic codes H16 (corneal ulceration) and H18 (other corneal disorders). Inclusion criteria for the study were the following: (1) a documented history of at least one episode of microbial keratitis, and (2) age ≥ 18 years.

Exclusion criteria encompassed cases of non-inflammatory keratitis (e.g., autoimmune, phototoxic, or toxic etiologies), recurrent corneal erosions, episcleritis, and other corneal abnormalities such as corneal dissection, keratoconus, and descemetocele (ICD-10 codes H16 or H18.7). Patients with hereditary corneal dystrophies, including epithelial basement membrane dystrophy, granular, reticular, macular corneal dystrophy, and Fuchs’ endothelial dystrophy (H18.5), were also excluded. Additional exclusion criteria included changes in corneal membranes of unknown origin (e.g., Descemet’s folds, cracks; H18.3), bullous keratopathy, blepharitis, chalazion, hordeolum, conjunctivitis, pterygium, pinguecula, and trachoma. Furthermore, patients with keratoplasty failure, classified under H18 (e.g., bullous keratopathy or other forms of corneal degeneration), were also excluded from the analysis.

Data on the following variables were collected:

(a) Sociodemographic characteristics: age, gender, and ophthalmological history;
(b) The number of previous keratitis episodes and time since the last occurrence;
(c) Time from symptom onset to emergency visit;
(d) Pre-visit treatment (ocular and systemic);
(e) Risk factors for keratitis, including contact lens (CL) use, type of CL, history of eye trauma, post-corneal transplant condition, and CL misuse behaviors (e.g., sleeping, swimming, or bathing with CLs, using tap water for cleaning, or prolonged CL use).

The data were collected on suspected etiology (e.g., viral, bacterial, fungal, acanthamoeba, or mixed infections), lesion location (central, paracentral, peripheral, entire surface), lesion multiplicity, concomitant uveitis, and visual acuity at admission, measured as logarithm of the minimum angle of resolution (logMAR). Paracentral location was defined as a diameter of more than 4 central and less than 8 mm peripheral cornea. Additionally, lens status was recorded to assess its potential confounding effect on visual acuity impairment associated with microbial keratitis.

The treatment approach was recorded, including ocular treatments (antibiotics, antivirals, antifungals, steroids, antiamoebics, and mixed treatments), systemic therapies, and additional ocular treatments such as lubricants, antiglaucoma medications, disinfectants, subconjunctival dexamethasone injections with gentamicin, intraocular methylprednisolone, anti-inflammatory agents, antiamoebics, and cycloplegia agents (e.g., tropicamide, atropine).

LogMAR thresholds for logistic regression were set at 0.3, which corresponds to moderate visual impairment according to the World Health Organization (WHO) definition. LogMAR ≤ 0.3 (equals 5/10 in Snellen charts) represents acceptable visual acuity for daily activities.

### 2.3. Definitions

- Naïve keratitis: a single episode of keratitis.
- Recurrent keratitis: a subsequent episode of keratitis occurring at least 3 months after the previous episode or within 3 months of complete healing.
- Primary keratitis: a keratitis that occurs after direct contact with the infectious agent in an individual without latent infection.
- Microbial keratitis: any inflammatory condition of cornea caused by a microorganism or infective agent (bacteria, fungi, protozoa, viruses, or prions), confirmed by positive scrape results or characteristic lesions observed via slit lamp examination with fluorescein staining.
- Therapeutic keratoplasty: a transplant performed for debulking and preserving globe integrity.
- Mild keratitis: corneal inflammation with reduced best-corrected visual acuity (BCVA) < 0.5 LogMAR.
- Moderate keratitis: corneal inflammation with BCVA ≥ 0.5 and <0.99 LogMAR.
- Severe keratitis: corneal inflammation with central or large peripheral ulcers with BCVA ≥ 1.0 LogMAR.
- CL misuse: defined as sleeping, swimming, or bathing with CLs, cleaning them with tap water, or prolonged use.
- Mixed initial diagnosis: suspected etiology involving at least two pathogens (virus, bacteria, fungus, or amoeba) based solely on clinical examination.
- Systemic treatment: oral or intravenous administration of medication.

### 2.4. Statistical Methods

Numeric variables were summarized using the median and interquartile range (IQR) due to their non-normal distribution. Nominal variables were presented as counts and percentages. The normality of distributions was assessed using the Shapiro–Wilk test, skewness, and kurtosis. Comparisons of numeric variables between groups were performed using the Kruskal–Wallis test, with pairwise comparisons adjusted using the Dunn test and Bonferroni correction when necessary. Nominal variables were compared using Pearson’s chi-square test or Fisher’s exact test, as appropriate. The Mann–Whitney U test was used to compare BCVA (logMAR) between the two groups.

Logistic regression analysis was employed to identify factors associated with logMAR ≤ 0.3. A cutoff of *p* = 0.250 was used for pre-selection of variables for the multivariate model, and stepwise selection was applied for the final model. Model fit was assessed using Nagelkerke R^2^ and the Hosmer–Lemeshow Goodness of Fit (GOF) test. Multicollinearity was checked using Variance Inflation Factors (VIFs). Statistical significance was set at *p* < 0.05. The analysis was carried out using Statistica software (version 13.3) (Table 1 and Table 2) and R statistical software (version 4.4.2) (Table 3 and Table 4).

## 3. Results

### 3.1. Demographics

This study included 1303 patients with keratitis, divided into two groups: 233 patients with recurrent keratitis (Group 1) and 1070 patients with naïve keratitis (Group 2). At baseline, there were no significant differences between the groups in terms of age (median age 51 years vs. 45 years, *p* = 0.989) and gender (61.80% vs. 57.85% female, *p* = 0.267) (Table 1). Significant differences were observed for the following: (1) the presence of refractive error, which was more common in Group 2 (4.72% in Group 1 vs. 11.68% in Group 2, *p* = 0.001), and (2) corneal transplantation history, which was more frequent in Group 1 (7.73% vs. 4.21%, *p* = 0.049) (Table 1). There were no differences in terms of cataract prevalence between the groups (0.86% vs. 1.21%, *p* = 0.640). The study population was predominantly Caucasian, comprising 100.00% (n = 233) of the recurrent keratitis group and 97.83% (n = 1066) of the non-recurrent group. Other reported ethnicities, including African and Asian, each accounted for less than 1% of the total sample.

### 3.2. Group Characteristics

Group 1 was characterized by a median best-corrected visual acuity (BCVA) of logMAR 0.30 (IQR: 0.08–1.00), with 96.14% of patients managed as outpatients and 12.02% being CL users (Table 1). The mean time from symptom onset to medical consultation was 17.09 ± 125.13 days (Table 2). Similarily, Group 2 also had a median BCVA of logMAR 0.30 (IQR: 0.08–0.90), but only 82.43% of patients were managed as outpatients and there was a significantly higher percentage of patients wearing CLs (30.28%, *p* = 0.000) (Table 1).

### 3.3. Initial Diagnosis

The average time from symptom onset to the first medical visit did not differ significantly between groups (Group 1: 17.09 ± 125.13 days; Group 2: 10.78 ± 27.39 days, *p* = 0.463). Lesion location also (divided into the following: central, midperiphery, periphery of the cornea) showed no significant differences between groups (*p* = 0.436), but multiple lesions were more common in recurrent keratitis (*p* = 0.001) (Table 2).

**Figure 1:**
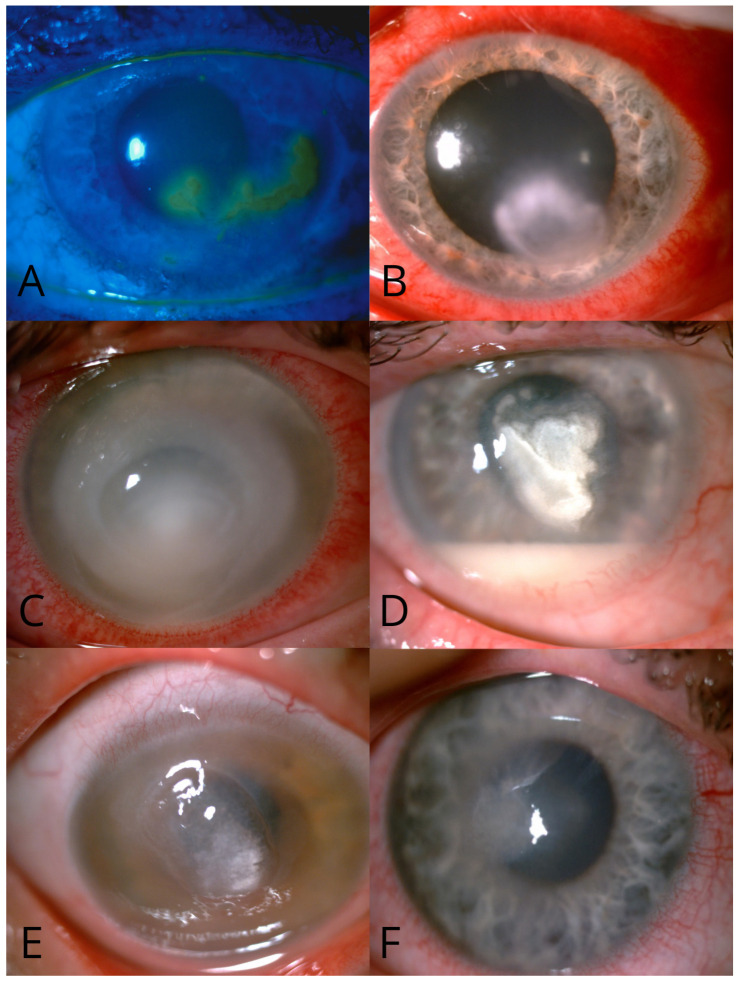
Representative clinical photographs illustrating the spectrum of microbial keratitis etiologies: (**A**) *Herpetic keratitis* caused by herpes simplex virus, showing characteristic dendritic corneal ulceration staining with fluorescein. (**B**) *Gram-positive bacterial keratitis* (*Staphylococcus haemolyticus*, *Staphylococcus epidermidis*) presenting with conjunctival injection, hyperemia, and a focal white corneal infiltrate. (**C**) *Gram-negative bacterial keratitis* due to *Pseudomonas aeruginosa*, with a mid-peripheral white corneal infiltrate, melting, and hypopyon. (**D**) *Fungal keratitis,* showing a gray-white stromal infiltrate with feathery borders, satellite lesions, and hypopyon. (**E**) *Acanthamoeba keratitis,* typically presenting with epitheliopathy, limbitis, and a ring-shaped stromal infiltrate. (**F**) *Mixed infection* with *Acanthamoeba* and *Candida albicans*, demonstrating features of both protozoal and fungal keratitis.

Initial diagnoses varied between groups. Direct bacterial etiology was more prevalent in Group 2 (47.11% vs. 38.09%), while viral etiology was more common in Group 1 (31.60% vs. 19.87%, *p* < 0.001). Fungal etiology constituted 4.1% of the whole cohort (54 cases). Mixed infections accounted for 10.05% of cases across the entire cohort (Table 2). Figure 1 presents representative images of corneal ulcers of various etiologies observed in our cohort.

### 3.4. Treatment Approaches

Topical antibiotics were the most frequently used treatment, applied in 90.18% of cases, followed by antivirals (23.94%), steroids (15.58%), and antifungal agents (14.89%). General (systemic) treatments were administered to 46.66% of patients, while 4.07% required an amniotic membrane transplant. Group 1 received topical antivirals significantly more often (35.49% vs. 21.46%, *p* < 0.001) and steroids (22.08% vs. 14.18%, *p* = 0.003) compared to Group 2. Amniotic membrane transplants were more common in Group 2 (4.76% vs. 0.87%, *p* = 0.007). The need for corneal transplants did not differ significantly between groups (30.30% vs. 24.72%, *p* = 0.078) (Table 2).

### 3.5. Visual Outcomes

**Figure 2:**
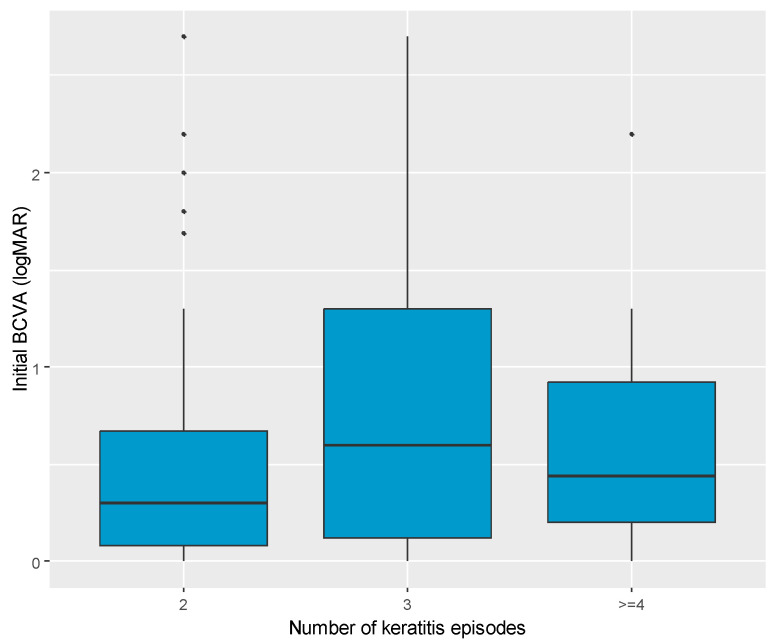
Boxplot charts presenting the distribution of median values of initial BCVA (logMAR) among patients with two episodes, three episodes, and four or more episodes of recurrent keratitis.

Among patients with recurrent keratitis, BCVA (logMAR) significantly differed based on the number of recurrences (*p* = 0.049) (Figure 2). Median BCVA was highest in patients with three episodes (logMAR 0.60), lower in those with four or more episodes (logMAR 0.44), and lowest in those with two episodes (logMAR 0.30), (Table 4).

**Figure 3:**
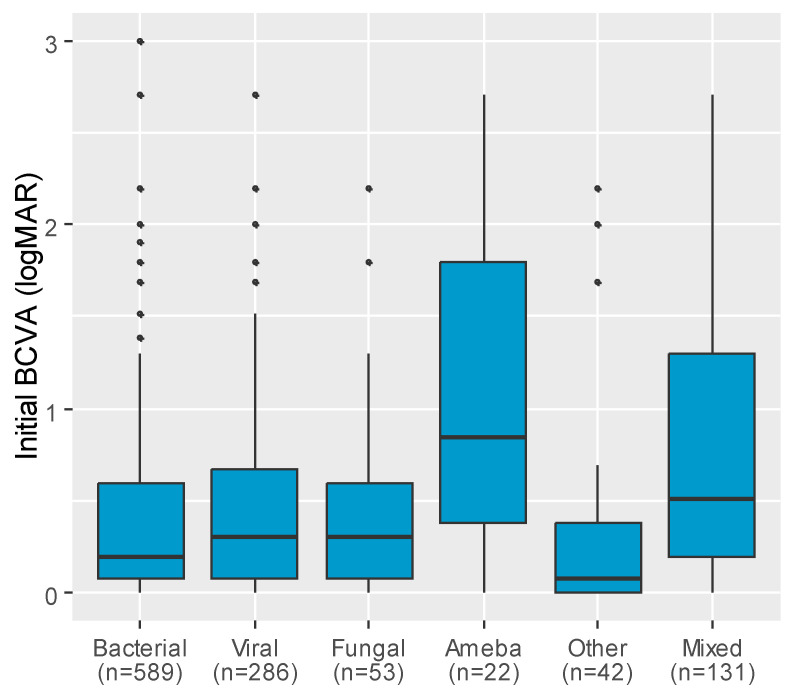
Boxplot chart presenting the distribution of median initial BCVA values (logMAR) among patient groups depending on etiology.

**Figure 4:**
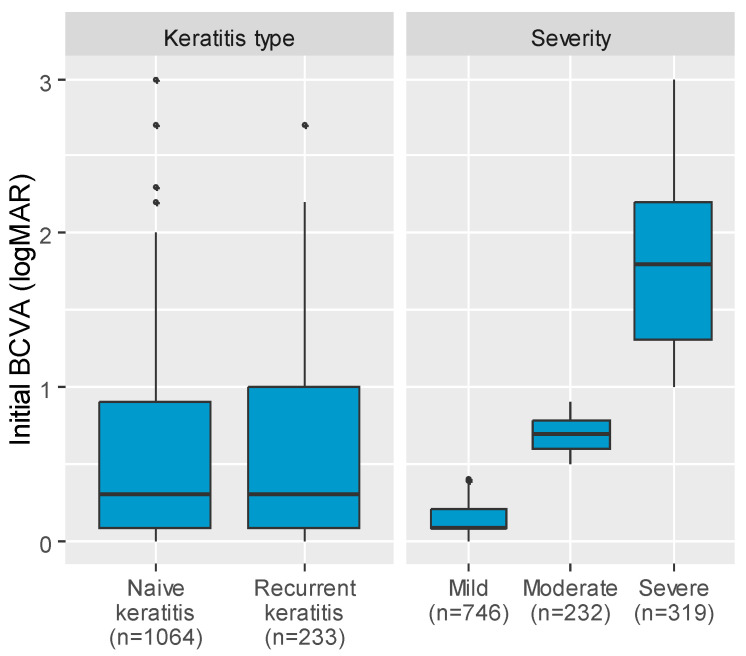
Boxplot charts illustrating the distribution of median initial BCVA (logMAR) values across patient groups, stratified by keratitis type and severity.

The median BCVA also differed significantly depending on the etiology of the infection (*p* < 0.001). The median BCVA was the lowest among patients with Acanthamoeba (logMAR 0.85), followed by those with mixed etiology (logMAR = 0.51), viral and fungal infection (logMAR 0.30 both groups), or bacterial infection (logMAR 0.20) (Figure 3). Post hoc analysis revealed that the median BCVA in patients with Acanthamoeba infection was significantly worse compared to other infection types, except mixed infections. Significant differences were also observed between viral and bacterial infections, viral and other infections, and between mixed and bacterial infections (Table 3). The median of the initial BCVA was comparable between the recurrent and naïve infection groups (Figure 4). Most patients presented with mild keratitis (n = 746), followed by severe cases (n = 319), and lastly, moderate keratitis (n = 232) (Figure 4).

Multiple lesions were observed to be significantly more common in patients with four or more recurrences (66.7%) compared to those with two or three episodes (30.3% and 33.3%, respectively, *p* = 0.010) (Table 4).

### 3.6. Prognostic Factors

Univariate logistic regression identified several factors associated with better visual prognosis (logMAR ≤ 0.3), including younger age (OR = 0.96, 95% CI: 0.96–0.97, *p* < 0.001), CL use (OR = 2.37, 95% CI: 1.84–3.08, *p* < 0.001), and appropriate CL usage (OR = 2.25, 95% CI: 1.42–3.66, *p* = 0.001) (Table 4).

In multivariate analysis, older age (OR = 0.97, 95% CI: 0.96–0.97, *p* < 0.001), longer time from symptom onset to consultation (OR = 0.99, 95% CI: 0.98–1.00, *p* = 0.008), and post-corneal transplant status (OR = 0.11, 95% CI: 0.02–0.32, *p* < 0.001) were significantly associated with worse prognosis. Interestingly, multiple lesions were linked to improved prognosis (OR = 1.82, 95% CI: 1.24–2.70, *p* = 0.002) (Table 5).

## 4. Discussion

Our study revealed a recurrence rate of 17.88% (233 out of 1303 patients), regardless of etiology. Most recurrent cases were managed in outpatient settings (224 patients (96.14%), while only a small fraction required hospitalization (9 patients, 3.86%). Patients experiencing three episodes of infection had the highest median logMAR (0.60 (IQR: 0.12–1.30) compared to those with two episodes (0.30 (IQR: 0.08–0.67, *p* = 0.049). Eyes that underwent corneal transplantation presented lower BCVA compared to those without corneal transplantation (OR = 0.11; 95% CI: 0.02–0.32; *p* < 0.001). BCVA deterioration also depended on the patient’s age; the multivariate analysis showed that every added year lowered the median logMAR (OR = 0.97; 95% CI: 0.96–0.97; *p* < 0.001). These findings prove that repeated episodes of keratitis pose a threat to vision health and lead to progressive vision deterioration. The first recurrence was associated with mild BCVA decline (logMAR 0.30); however, the second was with a twofold fall (logMAR 0.60). History of three relapses also diminished BCVA but less than two relapses that occurred to be the most detrimental for vision prognosis. We showed that keratitis recurrence usually leads to vision loss; however, the scale of deterioration remains independent of the number of recurrence episodes. We determined that the most independent factors contributing to vision loss in recurrence were age and previous keratoplasty. Keratitis recurrence was routinely treated in outpatient clinics, with singular cases requiring hospital care. Thus, relapses had rather a mild course and resolved without complications requiring a surgical approach.

The recurrence rates of keratitis vary widely in different studies, depending on the study design, observation time, underlying etiology, and treatment applied [24,25,26,27]. The risk of recurrence of herpesvirus keratitis (HSK) was assessed at 24.5% ± 4.5 and 32.9% ± 6.5 at one and two years and 65% at three years in prospective studies [24,27]. Labetoulle’s team revealed a similar rate of 58% using only 3 months of follow-up and calculated the estimated incidence as 18.3 per 100.000 person years among the French population [25]. The short observation period minimized the influence of seasonal variations; however, the multicenter design and the involvement of a large number of ophthalmologists (n = 412) in patient assessment may have increased the risk of diagnostic variability or misclassification [25]. In a 16-year retrospective study by Kaye et al., the recurrence rate of bacterial keratitis, confirmed via microbiological analysis, was reported at 12.15% among 2418 participants [28]. The recurrence rate of 17.88% observed in our study is more consistent with that reported for bacterial keratitis, which may be attributed to the higher number of bacterial cases compared to viral ones in the cohort (593 vs. 286). However, our findings indicate a notable trend: viral infections were significantly more likely to be associated with recurrent episodes of keratitis (31.33% vs. 19.91%, *p* = 0.001), whereas bacterial infections more frequently accounted for first-time episodes (47.00% vs. 38.63%, *p* = 0.001). This suggests that viruses, in contrast to bacteria, are more commonly implicated in recurrent infections—a pattern likely driven by differing relapse mechanisms. In particular, most cases of herpetic stromal keratitis (HSK) represent reactivations of latent infections, while primary HSK episodes occur less frequently [29]. Viral pathogens, particularly herpesviruses, persist in a latent state within sensory ganglia neurons and may reactivate in response to immune suppression or systemic stress, leading to recurrent episodes. In contrast, bacterial recurrences are often associated with the colonization of adjacent ocular structures, such as the eyelid margins and conjunctiva. Notably, endogenous carriers of *Staphylococcus aureus* or *Pseudomonas aeruginosa* exhibit higher rates of keratitis relapse compared to non-carriers, suggesting that persistent colonization serves as a reservoir for reinfection [28,30,31,32]. However, HSK can closely mimic infections caused by varicella-zoster virus (VZV) or *Acanthamoeba* spp., making accurate differentiation challenging. Fortunately, important differences exist between these two pathogens in terms of clinical signs and symptoms, underlying mechanisms, and disease progression. When correctly recognized, these distinctions can support a more accurate initial diagnosis. Herpes simplex keratitis (HSK) may affect multiple layers of the cornea, including the epithelium, stroma, and endothelium [33]. Epithelial HSK typically presents with dendritic or geographic ulcers that stain with fluorescein. This appearance can resemble early-stage (less than 1 month of symptoms) Acanthamoeba keratitis (AK), which may also feature pseudodendritic ulcers, potentially complicating the initial diagnosis [34]. However, key differences may help distinguish between the two. A hallmark of HSK infection is reduced corneal sensation, whereas AK is usually associated with significant pain from the onset—often out of proportion to the clinical signs [35]. When the stroma is involved, the clinical signs become even more distinctive. In stromal HSK, the epithelium typically remains intact [36]. In contrast, in AK, stromal involvement occurs at a later stage following epithelial inflammation when epithelial loss is usually evident. The study by Dart et al. found that the most common clinical features of early-stage AK are punctate keratitis and perineural infiltrates, with limbitis being especially prominent [35]. Notably, limbitis occurs with similar frequency in both the early and late stages of AK and is strongly associated with another characteristic feature of the disease—CL use and its misuse, as 93–95% of AK cases occur in CL users [37]. As the condition progresses, a key feature that distinguishes AK from HSK is the appearance of a characteristic paracentral ring infiltrate. Recognizing such subtle yet important differences can lead to an earlier and more accurate diagnosis—before culture results are available—and allow for the prompt initiation of appropriate treatment. Advanced diagnostic tools such as polymerase chain reaction (PCR), in vivo confocal microscopy, or next-generation sequencing may be essential for achieving definitive diagnosis and improving diagnostic accuracy [38,39,40,41,42]. This study highlights the limited access to PCR and genetic testing at our center. As a result, corneal scraping followed by microbiological analysis remains the primary method for confirming infection. However, the retrospective design of the study limited the availability of complete culture data—particularly in patients who were referred after starting empirical treatment, at which point cultures were no longer viable for analysis. Additionally, many patients admitted through the emergency department did not return for follow-up visits, further hindering the ability to adjust treatment based on culture results. Despite these challenges, our findings reinforce that clinical assessment, particularly a thorough patient history, remains essential in the absence of molecular diagnostics. This underscores the urgent need to incorporate PCR and other molecular tools into routine ophthalmic practice to improve diagnostic accuracy, guide more personalized treatment, and reduce the risk of recurrence or complications [8,38].

Last, but not least, fungal keratitis warrants particular attention. Although its incidence among recurrent cases was relatively low (17/233), it was frequently associated with poor visual outcomes (logMAR > 0.3; OR = 0.39; 95% CI: 0.25–0.58; *p* < 0.001). This might be partly attributed to the higher rate of complications and delayed healing compared to bacterial ulcers. Notably, 25–35% of acute fungal ulcers require surgical intervention, with evisceration rates reported as high as 30% [39,40]. It is crucial to emphasize the importance of performing corneal scraping and microbiological analysis, including susceptibility testing, to optimize empirical treatment and improve clinical outcomes. However, the retrospective nature of this study limited our ability to retrieve complete corneal culture results. Furthermore, most patients were referred to our tertiary center with corneal ulcers that were already being treated empirically. As a result, corneal cultures were not routinely performed at the time of presentation due to the ongoing administration of broad-spectrum antimicrobial therapy. However, due to the low sensitivity of microbiological cultures for detecting fungal pathogens, and the fact that post-transplant keratitis patients typically receive multiple antimicrobial therapies, it is difficult to fully assess this impact in a retrospective study.

A common and modifiable predisposing factor in MK is CL use, which is mostly associated with bacterial infections, particularly with Pseudomonas aeruginosa [28,41]. A CL, lying on the corneal surface, limits access to oxygen, which impairs corneal metabolism, cell proliferation, migration, and exfoliation, predisposing the cornea to bacteria adhesion [42,43,44,45]. CLs contribute to bacterial infection by disrupting biofilm balance—while ocular microbiota is predominantly aerobic, hypoxia promotes anaerobic bacteria growth [46]. Sankaridurg et al. and Keay et al. revealed that besides the cornea, a hydrogel CL itself poses a substrate for bacteria, leading to infiltrative keratitis [47,48]. Despite new CL types (silicone hydrogel, a highly oxygen-permeable soft CL), the rate of infection associated with CLs remains constant [46]. In our study, CL wearers tend to have rather naïve keratitis than recurrent. We found the same association in terms of CL misuse. Mucci et al. revealed the opposite correlation—CL wearers had a higher median recurrence rate (0.4 episodes/year) in contrast to non-CL wearers (0.2 episodes/year) (*p* = 0.02) [49]. The study was retrospective within 4 years of observation; however, the number of enrolled patients was relatively small (n = 117). The counterintuitive effect in our study is unlikely due to the lenses themselves but rather to behavioral changes—the possibility of discontinuing CL wear after the first episode of keratitis among the naïve group. Future prospective studies may answer how relevant CL withholds are to prevent recurrence. Several previous studies focused only on risk factors identification—omitting the question of how they correlate with the infection’s severity and finally—how they affect the patient’s vision condition. The severity of the disease is usually measured as the rate of vision reduction, lesion localization (central or large peripheral ulcers), or by the cost and duration of a disease. We classify keratitis as mild (considered sufficient for daily activities) if logMAR is even or less than 0.3 [50]. In our study, CL-related keratitis was statistically mild, while CL wearers presented mainly with logMAR ≤ 0.3 suggesting the protective effective of CL use on BCVA. We hypothetize that our CL wearers were more knowledgeable about the risks associated with CL use and more aware of keratitis symptoms than CL non-wearers, which is consistent with Alzahrani et al., who showed that current CL users were more aware of safety practices compared to previous users (92% vs. 81.2%, *p* < 0.05) [51]. MK affects approximately 5 out of 10,000 CL wearers and mostly stems from improper hygiene practices like overnight or extended use [52,53]. Our results suggest that CL wearers were well educated about possible complications and followed the given guidelines.

Beyond etiology and established risk factors, visual outcomes in microbial keratitis (MK) are influenced by several additional variables, including age, lesion characteristics, treatment modality, and the timing of treatment initiation. Among these, lesion morphology—particularly lesion multiplicity and location—was found to play a critical role in visual prognosis. Our findings indicate that multiple peripheral lesions, which typically spare the central visual axis, are associated with more favorable visual outcomes compared to singular central lesions that directly compromise the macular area. Clinically, multiple lesions are often perceived as more complex and are consequently managed more aggressively from the outset, which may contribute to improved best-corrected visual acuity (BCVA) [54].

In our study, pre-visit treatment was neutral to BCVA outcomes. However, the need for systemic treatment at admission (oral or intravenous) correlated with a worse BCVA, likely because systemic therapy is usually reserved for severe cases. The complexity of applied therapy depends on the clinical stage of the disease, patients’ symptoms, and infection etiology. In more severe cases, amniotic membrane transplant (AMT) can be used as an adjunctive procedure for corneal reconstruction. AMT promotes rapid epithelialization and minimizes neovascularization—a potential risk factor for keratitis recurrence, which contributes to faster corneal healing and better visual outcomes [55]. In our study, AMT was more often performed among the recurrent keratitis group than with naïve inflammation (2 (0.86%) vs. 51 (4.77%), *p* = 0.006), which suggests that recurrent cases were more severe and needed more complex treatment. However, treatment effectiveness depends not only on the method used but also its timing. Any delay in patients reporting to the eye care center allows the infection to evolve, decreasing the patient’s prognosis and options for less invasive therapies [54,56]. Hoffman et al.’s study demonstrated that MK patients with a longer median time from symptom onset to presentation (8 vs. 5 days, *p* < 0.001) had worse BCVA (0.6 vs. 0.3 logMAR, *p* < 0.001) [57]. In our study, we observed a similar correlation that longer intervals between first symptoms and treatment correlated with worse logMAR outcomes (OR = 0.99; 95% CI: 0.98–1.00; *p* = 0.008, days). Recent reports also showed that older age (>50 years old) was a significant indicator for poor visual outcomes (CDVA logMAR > 0.6) (OR = 2.61; 95% CI: 1.24–5.47; *p* = 0.011) and poor corneal healing (>30 days) (OR = 1.86; 95% CI: 1.06–3.24; *p* = 0.030) [23]. Increased permeability of the cornea and reduced number of endothelial cells, poorer lacrimal drainage, eyelid malposition disorders (entropion, ectropion), and reduction in corneal sensitivity are common factors that expose elders to greater risk of MK [58,59,60]. The cornea is also a hormone-responsive tissue and is particularly sensitive to age-related hormonal changes in women. After menopause, decreased levels of estrogen and progesterone are associated with central corneal thinning and increased symptoms of dry eye. Both of these changes can weaken the barrier, making the eye more susceptible to infection [61]. Our findings are consistent with previous studies, demonstrating that older age is significantly associated with worse best-corrected visual acuity (BCVA). In our study, females were more likely to experience recurrent keratitis than a first-time infection, which may be explained by the higher average age in the recurrent group and the impact of postmenopausal hormonal changes. However, even females of reproductive age may be more vulnerable to corneal infection [62]. The cyclical rise in estrogen at the beginning of the menstrual cycle promotes water retention in the cornea, increasing central corneal thickness. These hormonal fluctuations can alter stromal collagen structure and matrix metalloproteinase activity, reducing the biomechanical stability of the cornea and potentially facilitating microbial infection [63]. Additionally, a longer interval between symptom onset and clinical presentation, advanced patient age, and fungal etiology were all linked to more severe visual impairment. Moreover, a pronounced inflammatory response may result in corneal perforation, which poses a substantial risk for the intraocular spread of infection and the development of panophthalmitis. In such cases, surgical intervention is often required, with evisceration sometimes being unavoidable [64].

Keratoplasty serves as the final treatment option for MK cases that are unresponsive to antimicrobial therapy or complicated by corneal perforation. However, any surgical intervention introduces mechanical trauma to ocular tissues, and keratoplasty—regardless of type—carries a risk of MK recurrence. Although relatively uncommon, such recurrences represent a serious complication that can significantly compromise best-corrected visual acuity. The MK recurrence rate after therapeutic penetrating keratoplasty (TPK) varies from 6.34% to 31.6% [1,65,66,67,68,69,70]. Lamellar techniques, such as DALK, achieved better outcomes in the Wu et al. study. However, a 4-year observation study showed a similar recurrence rate to TPK—33% [71,72]. In our study, patients who had undergone keratoplasty experienced more frequent naïve keratitis compared to recurrence (18 cases [7.73%] vs. 45 cases [4.21%], *p* = 0.023). It is important to highlight that not all cases within the naïve group are the result of direct pathogen exposure. Many recurrences in post-keratoplasty patients are attributed to the reactivation of latent infections, often triggered by immunosuppressive therapy used to prevent graft rejection [73]. Our data, drawn from a tertiary referral center, may be biased toward more severe keratitis cases, which are often treated with systemic immunosuppression. This could lead to an overestimation of latent infection reactivation classified as naïve cases. Notably, a history of keratoplasty before MK significantly lowered the chance for better BCVA (logMAR ≤ 0.3) during keratitis episodes either in the univariate (OR = 0.04; 95% CI: 0.01–0.11; *p* < 0.001) or multivariate regression analysis (OR = 0.11; 95% CI: 0.02–0.32; *p* < 0.001). This aligns with other studies’ outcomes. Overall, 59–78.4% of patients after post-keratoplasty MK achieved BCVA logMAR > 1, regardless of etiology [69,74]. Severe visual deterioration is frequently a consequence of corneal scarring or graft failure secondary to recurrent inflammation. One of the older surgical approaches, therapeutic penetrating keratoplasty (TPK), remains effective in eradicating microbial keratitis, but finally fails in 46–60.7% of grafts [65,75]. The observed decline in best-corrected visual acuity (BCVA) associated with keratoplasty-related recurrence underscores the importance of patient education and early identification of post-transplant complications in optimizing visual outcomes. Enhancing awareness of corneal infection symptoms, encouraging prompt presentation to ophthalmic care centers, and advancing diagnostic capabilities may collectively reduce the need for corneal transplantation and significantly improve patient prognosis.

## 5. Study Limitations

This study has several limitations. Firstly, data collection was retrospective and conducted at a single center, which may limit the generalizability of the findings. Secondly, visual acuity was assessed only at the time of admission, preventing the evaluation of long-term visual outcomes. Furthermore, the absence of standardized diagnostic criteria and a definitive gold standard for microbial keratitis complicates the differentiation between true infections, contamination, and coexisting pathogens, potentially impacting diagnostic accuracy.

## 6. Conclusions

Our findings suggest that recurrent episodes of microbial keratitis are often non-urgent and can be managed without the need for invasive interventions. Although each recurrence contributes to cumulative vision loss, the frequency of recurrence did not correlate with the magnitude of best-corrected visual acuity decline. Visual outcomes were significantly influenced by factors such as advanced age, fungal etiology, delayed clinical presentation, and a history of corneal transplantation, all of which were associated with a logMAR increase exceeding 0.3. Notably, contact lens use was not linked to poorer visual outcomes or a higher risk of recurrence. Nonetheless, prospective studies are warranted to validate these findings and to address the inherent limitations of our retrospective design.

## Figures and Tables

**Table 1:** Demographic and clinical characteristics of patients with recurrent (Group 1, n = 233) and non-recurrent keratitis (Group 2, n = 1070).

Variable	Group 1 (Recurrent Keratitis)	Group 2 (Non-Recurrent Keratitis)	*p*
N	233	1070	
Age, years, median (IQR)	51.00 (36.00; 68.00)	45.00 (31.00; 67.00)	0.989 ^2^
Sex, female, n (%)	144 (61.80)	619 (57.85)	0.267 ^1^
Ethnicity, n (%)			
Caucasian	233 (100.00)	1066 (97.83)	0.584 ^1^
African	-	2 (0.19)	1.00 ^1^
Asian	-	2 (0.19)	1.00 ^1^
Eye condition, (n %)	2 (0.86)	13 (1.21)	0.644 ^1^
Cataract, n (%)	11 (4.72)	125 (11.68)	**0.001 ^1^**
Refractive error, n (%)	18 (7.73)	45 (4.21)	**0.023 ^1^**
Post-corneal transplant, n (%)			
PK	3 (0.01)	31 (0.03)	0.242 ^1^
DALK	1 (0.004)	4 (0.004)	1.00 ^1^
DSAEK	1 (0.004)	2 (0.002)	1.00 ^1^
Initial BCVA (logMAR), median (IQR)	0.30 (0.08; 1.00)	0.30 (0.08; 0.90)	0.502 ^2^
Management type, n (%)			
Inpatient, n (%)	9 (3.86)	188 (17.57)	**0.000 ^1^**
Outpatient, n (%)	224 (96.14)	882 (82.43)	
CL use, n (%)	28 (12.02)	324 (30.28)	**0.000 ^1^**
CL misuse *, n (%)	5 (2.15)	85 (7.94)	**0.002 ^1^**
History of ocular trauma, n (%)	10 (4.29)	127 (11.87)	**0.001 ^1^**

IQR—interquartile range. Nominal variables compared with Pearson’s chi-square test ^1^ or Fisher’s exact test, as appropriate. Numeric variables compared with Mann–Whitney U test ^2^, bold indicates statistically significant *p*-values. * Defined as sleeping, swimming, or bathing while wearing the CL, cleaning the CL with tap water, or prolonged CL use. PK—penetrating keratopathy, DALK—Deep Anterior Lamellar Keratopathy, DSAEK—Descemet Stripping Automated Endothelial Keratoplasty.

**Table 2:** Clinical features, initial diagnosis, and treatment details of patients with recurrent and non-recurrent keratitis.

Variable	Group 1 (Recurrent Keratitis)	Group 2 (Non-Recurrent Keratitis)	*p*
N	233	1070	
Time from the symptoms to the visit, days, mean (SD)	17.01 (±124.56)	10.79 (±27.42)	0.759 ^2^
**Clinical features in slit lamp test, n (%)**			
Central location, n (%)	47 (20.17)	248 (23.17)	0.356 ^1^
Paracentral location, n (%)	32 (13.73)	172 (16.07)	
Peripheral location, n (%)	101 (43.35)	461 (43.08)	
Total corneal surface area, n (%)	42 (18.03)	144 (13.46)	
Surface area, n (%)			
Singular lesion, n (%)	148 (63.52)	802 (74.95)	**0.000 ^1^**
Multiple lesions, n (%)	80 (34.33)	252 (23.55)	
**Initial diagnosis, n (%)**			
Direct bacteria, n (%)	90 (38.63)	503 (47.0)	**0.001 ^1^**
Direct virus, n (%)	73 (31.33)	213 (19.91)	
Direct fungi, n (%)	17 (7.3)	37 (3.46)	
Direct amoeba, n (%)	2 (0.86)	20 (1.87)	
Mix diagnosis, n (%)	30 (12.88)	101 (9.44)	
Initial treatment, n (%) *			
Antibiotic, n (%)	200 (85.84)	975 (91.12)	**0.024 ^1^**
Antiviral, n (%)	82 (35.19)	230 (21.5)	**0.000 ^1^**
Steroid, n (%)	51 (21.89)	152 (14.21)	**0.003 ^1^**
Antifungal, n (%)	26 (11.16)	168 (15.70)	0.078 ^1^
General treatment, n (%)	117 (50.21)	491 (45.89)	0.162 ^1^
Amniotic membrane transplant, n (%)	2 (0.86)	51 (4.77)	**0.006 ^1^**
Treatment before the visit, started by another clinician, n (%)	71 (30.47)	264 (24.67)	0.066 ^1^
Need for a corneal transplant, n (%)	1 (0.43)	47 (4.39)	0.509 ^1^

SD—standard deviation. Nominal variables compared with Pearson’s chi-square test ^1^ or Fisher’s exact test, as appropriate. Numeric variables compared with Mann–Whitney U test ^2^, bold indicates statistically significant *p*-values. * Each type of treatment considered as separate variable; thus, sum of all treatments exceeded 100% due to possibility of treatment combination per patient. Bold indicates statistically significant *p*-values.

**Table 3:** Comparison of median initial BCVA between patient groups based on etiology.

Variable	n	Initial BCVA (logMAR), Median (IQR)	*p*
Etiology			**<0.001**
Bacterial	589	0.20 (0.08; 0.60) ^abg^	
Viral	286	0.30 (0.08; 0.67) ^aceh^	
Fungal	53	0.30 (0.08; 0.60) ^d^	
Ameba	22	0.85 (0.38; 1.80) ^bcdf^	
Other	42	0.08 (0.00; 0.38) ^efi^	
Mixed	131	0.51 (0.20; 1.30) ^ghi^	

IQR—interquartile range. Comparison made with Kruskal–Wallis’s test. bold indicates statistically significant *p*-values. ^a–i^—outcomes of post hoc evaluation made with Dunn test with Bonferroni adjustment (^a^: *p* adj = 0.017, ^b^: *p* adj < 0.001, ^c^: *p* adj = 0.014, ^d^: *p* adj = 0.044, ^e^: *p* adj = 0.031, ^f^: *p* adj < 0.001, ^g^: *p* adj < 0.001, ^h^: *p* adj = 0.003, ^i^: *p* adj < 0.001).

**Table 4:** Clinical characteristics of patients with recurrent keratitis, stratified by the number of recurrence episodes (2 vs. 3 vs. ≥4).

Variable	Number of Keratitis Episodes			*p*
	2	3	≥4	
N	126	27	18	-
Age, years, median (IQR)	50.00 (33.25; 67.00)	57.00 (36.50; 68.50)	50.50 (43.25; 68.75)	0.397
Initial BCVA (logMAR), median (IQR)	0.30 (0.08; 0.67)	0.60 (0.12; 1.30)	0.44 (0.20; 0.92)	**0.049**
CL use, n (%)	21 (16.7)	1 (3.7)	1 (5.6)	0.141
CL misuse, n (%)	3 (2.4)	1 (3.7)	0 (0.0)	0.709
Refractive error, n (%)	3 (2.4)	3 (11.1)	0 (0.0)	0.080
Post-corneal transplant, n (%)	8 (6.3)	3 (11.1)	1 (5.6)	0.544
Time from the last keratitis, days, median (IQR)	382.00 (183.00; 1095.00)	730.00 (365.00; 1095.00)	776.50 (379.75; 2828.75)	0.117
Time from the symptoms to the visit, days, median (IQR)	3.00 (1.00; 5.00)	3.00 (2.00; 5.50)	3.00 (1.00; 4.75)	0.876
Treatment prior to emergency department visit, n (%)	37 (29.4)	4 (14.8)	5 (27.8)	0.301 ^1^
Initial treatment, n (%) *				
Antibiotic, n (%)	105 (84.0)	25 (92.6)	17 (94.4)	0.393
Antiviral, n (%)	48 (38.1)	11 (40.7)	3 (16.7)	0.182 ^1^
Steroid, n (%)	22 (17.5)	8 (29.6)	5 (27.8)	0.261 ^1^
Antifungal, n (%)	13 (10.3)	3 (11.1)	4 (22.2)	0.352
Other treatment, n (%)	65 (51.6)	14 (51.9)	12 (66.7)	0.481 ^1^
General treatment, n (%)	62 (49.2)	16 (59.3)	9 (50.0)	0.636 ^1^
Clinical features in slit lamp test, n (%)				
Central location, n (%)	28 (22.2)	3 (11.1)	2 (11.1)	0.226
Paracentral location, n (%)	19 (15.1)	2 (7.4)	2 (11.1)	
Peripheral location, n (%)	58 (46.0)	13 (48.1)	7 (38.9)	
Total corneal, n (%)	17 (13.5)	8 (29.6)	5 (27.8)	
Not indicated, n (%)	4 (3.2)	1 (3.7)	2 (11.1)	
Surface area, n (%)				
Singular lesion, n (%)	85 (69.7)	18 (66.7)	6 (33.3)	**0.010 ^1^**
Multiple lesions, n (%)	37 (30.3)	9 (33.3)	12 (66.7)	
Need for a corneal transplant, n (%)	1 (0.8)	0 (0.0)	0 (0.0)	>0.999

IQR—interquartile range. Numeric variables compared with Kruskal–Wallis’s test. Nominal variables compared with Pearson’s chi-square test ^1^ or Fisher’s exact test, as appropriate. bold indicates statistically significant *p*-values. * Each type of treatment considered as separate variable; thus, sum of all treatments exceeded 100% due to possibility of treatment combination per patient.

**Table 5:** Logistic regression analysis of factors associated with improved visual prognosis (logMAR ≤ 0.3), presented as both univariate and multivariate models.

Variable	Univariate Models			Multivariate Model		
	OR	95% CI for OR	*p*	OR	95% CI for OR	*p*
Age, years	0.96	0.96–0.97	**<0.001**	0.97	0.96–0.97	**<0.001**
Sex, male (vs. female)	1.05	0.84–1.31	0.672	-	-	-
CL	2.37	1.84–3.08	**<0.001**	-	-	-
CL misuse	2.25	1.42–3.66	**0.001**	-	-	-
Recurrent keratitis	0.98	0.74–1.30	0.899	-	-	-
Number of keratitis	0.77	0.53–1.03	0.120	-	-	-
Time from the last keratitis, days	1.00	1.00–1.00	**0.023**	-	-	-
Treatment prior to emergency department visit	0.82	0.64–1.05	0.120	0.80	0.59–1.09	0.158
Time from the symptoms to the visit, days	0.98	0.97–0.99	**<0.001**	0.99	0.98–1.00	**0.008**
Initial diagnosis						
Viral (vs. bacterial)	0.64	0.48–0.86	**0.003**	-	-	-
Fungal (vs. bacterial)	0.78	0.44–1.41	0.406	-	-	-
Amoeba (vs. bacterial)	0.15	0.05–0.39	**<0.001**	-	-	-
Other/Mix (vs. bacterial)	0.47	0.34–0.67	**<0.001**	-	-	-
Any treatment (vs. no treatment)	18.37	3.73–332.05	**0.005**	8.02	1.05–179.39	0.089
Antibiotic treatment (vs. no antibiotics)	0.89	0.61–1.30	0.555	-	-	-
Antiviral treatment (vs. no antiviral treatment)	0.97	0.75–1.25	0.814	-	-	-
Steroid treatment (vs. no steroids)	0.91	0.67–1.23	0.526	-	-	-
Antifungal treatment (vs. no antifungal treatment)	0.35	0.25–0.48	**<0.001**	0.39	0.25–0.58	**<0.001**
Other treatment (vs. not treated with other type of drugs)	0.98	0.79–1.23	0.873	-	-	-
General treatment (vs. no general treatment)	0.36	0.28–0.45	**<0.001**	-	-	-
Refractive error	0.87	0.61–1.24	0.445	-	-	-
Cataract	0.14	0.02–0.49	**0.009**	0.33	0.04–1.67	0.212
Post-corneal transplant	0.04	0.01–0.11	**<0.001**	0.11	0.02–0.32	**<0.001**
Paracentral location (vs. central)	2.25	1.56–3.25	**<0.001**	-	-	-
Peripheral location (vs. central)	4.70	3.48–6.39	**<0.001**	-	-	-
Total corneal location (vs. central)	1.48	1.01–2.16	**0.045**	-	-	-
Location not indicated (vs. central)	1.97	1.06–3.65	**0.032**	-	-	-
Multiple lesions	1.33	1.04–1.72	**0.026**	1.82	1.24–2.70	**0.002**

OR—odds ratio, CI—confidence interval, bold indicates statistically significant *p*-values.

## Data Availability

Available from the corresponding author upon reasonable request.
